# Genetic aspects of human prion diseases

**DOI:** 10.3389/fneur.2022.1003056

**Published:** 2022-10-05

**Authors:** Brian S. Appleby, Shashirekha Shetty, Mohamed Elkasaby

**Affiliations:** ^1^Department of Pathology, National Prion Disease Pathology Surveillance Center, Case Western Reserve University, Cleveland, OH, United States; ^2^Department of Neurology, University Hospitals Cleveland Medical Center/Case Western Reserve University, Cleveland, OH, United States; ^3^Department of Pathology, Center for Human Genetics Laboratory, University Hospitals Cleveland Medical Center/Case Western Reserve University, Cleveland, OH, United States

**Keywords:** prion disease, Creutzfeldt-Jakob disease, Gerstmann-Sträussler Scheinker disease, fatal familial insomnia, genetic Creutzfeldt–Jakob disease, genetics, prion protein, *PRNP*

## Abstract

Human prion diseases are rapidly progressive and fatal neurodegenerative conditions caused by a disease-causing isoform of the native prion protein. The prion protein gene (*PRNP*) encodes for the cellular prion protein, which is the biological substrate for prion disease transmission and neurotoxicity. Human prion diseases have three etiologies: sporadic, genetic, and acquired. *PRNP* polymorphisms and pathogenic variants play a large role in the frequency, age at onset, and clinicopathologic phenotype of prion diseases. Genetic prion diseases will be covered in detail and information necessary for clinical care, predictive genetic testing, and genetic counseling will be reviewed. Because the prion protein is necessary for transmission and neurotoxicity, many experimental treatments targeting its production are being investigated and hold potential promise as a disease modifying treatment for all forms of prion disease, including asymptomatic mutation carriers. This article will review genetic aspects of human prion disease and their influence on epidemiology, clinicopathologic phenotype, diagnostics, clinical management, and potential treatment approaches.

## Introduction

Prion diseases are rapidly progressive neurodegenerative conditions that are invariably fatal. Disease causing prions (PrP^D^) cause neurotoxicity and employ template directed protein misfolding to convert normal cellular prion protein (PrP^c^) into more PrP^D^. PrP^c^ is necessary for neurotoxicity and for disease propagation (1). Prion protein gene (*PRNP*) knock-out mice are resistant to developing scrapie when they are intracerebrally inoculated (2). Similarly, *PRNP* knockout mice are unable to propagate the disease, demonstrating that PrP^c^ is necessary for the clinical manifestation of disease and its transmission (3). Prion diseases affect a variety of animal species such as scrapie in sheep and goat, bovine spongiform encephalopathy in cattle, and chronic wasting disease in cervids.

Prion diseases also affect humans, most commonly in the form of Creutzfeldt-Jakob disease (CJD). CJD has an annual incidence of 1–2 new cases per million individuals worldwide (4). In the U.S., 1 in 6,239 deaths are due to prion disease and incidence is highest in older age groups (5). There are three etiologies for human prion disease: sporadic, genetic, and acquired. Most human prion diseases are sporadic (85%), followed in frequency by genetic (10–15%) and acquired prion diseases from consumption of contaminated beef, cadaveric hormones, cadaveric tissue transplants/grafts, or contaminated neurosurgical instrumentation (~1%). Sporadic prion diseases include sporadic CJD, sporadic fatal insomnia (sFI), and variably protease sensitive prionopathy (VPSPr). Genetic prion diseases include genetic CJD (gCJD), Gerstmann-Straussler-Scheinker Syndrome (GSS), and fatal familial insomnia (FFI). Acquired prion diseases include kuru, iatrogenic CJD (iCJD), and variant CJD (vCJD).

Diagnosis of human prion disease is achieved by recognizing the clinical syndrome, ruling out other potential etiologies, and using certain diagnostic tests that can be suggestive of prion disease. Cerebrospinal fluid (CSF) testing is often used to diagnose prion disease. The most specific CSF test is real time quaking induced conversion (RT-QuIC), which amplifies and detects abnormal prion protein seeding. Across sporadic and genetic prion diseases, CSF RT-QuIC has a diagnostic sensitivity of 90% and specificity of 99% (6). Other less specific CSF surrogate markers, including 14-3-3 and total tau proteins, are also sometimes used in diagnosis (7). Brain magnetic resonance imaging (MRI) is also useful and has a diagnostic sensitivity of 70–95% and specificity of approximately 98% for sporadic CJD depending on the criteria (8). A diagnosis of genetic prion disease is made through the detection of a pathogenic *PRNP* variant and iatrogenic CJD is diagnosed through recognition of known acquired prion disease risk factors including cadaveric human growth hormone, dura mater grafts, corneal transplants, and contaminated neurosurgical instrumentation (9). Variant CJD is diagnosed by clinical syndrome, potential exposure history, and can be aided by the presence of the pulvinar sign on brain MRI (10). The only way to definitively diagnose prion disease and determine its type is through neuropathologic examination; however, antemortem diagnosis can be reliably achieved with the current diagnostic modalities mentioned above (11, 12) (Table 1).

**Table 1:** Diagnostic criteria for Creutzfeldt-Jakob disease (CJD) (12).

Definite sporadic CJD	• Diagnosed by standard neuropathologic techniques (e.g., protease-resistant prion proteins on immunohistochemistry and/or western blot)
Probable Sporadic CJD	• Neuropsychiatric disorder plus positive real time quaking induced conversion (RT-QuIC) testing -OR- • Rapidly progressive dementia with at least two of the following: ∘ Myoclonus ∘ Visual and/or cerebellar signs ∘ Pyramidal and/or extrapyramidal signs ∘ Akinetic mutism -AND- • One of the following positive test results: ∘ Periodic sharp wave complexes on electroencephalogram ∘ Positive 14-3-3 cerebrospinal fluid test with a disease duration of < 2 years ∘ High signal in caudate/putamen or at least two cortical regions (temporal, parietal, occipital) on DWI or FLAIR sequences of a brain MRI -AND- • Other investigations exclude an alternative diagnosis
Possible Sporadic CJD	• Progressive dementia and at least two of the following: ∘ Myoclonus ∘ Visual or cerebellar signs ∘ Pyramidal or extrapyramidal signs ∘ Akinetic mutism • Duration of illness < 2 years • Other investigations exclude an alternative diagnosis
Iatrogenic CJD	Progressive cerebellar syndrome in a recipient of human cadaveric-derived pituitary hormone; or CJD with a recognized acquired prion disease exposure risk
Familial CJD	Definite or probable CJD in a patient with definite or probable CJD in a first degree relative; and/or symptoms of CJD plus a disease-specific *PRNP* mutation

DWI, diffusion weighted imaging; FLAIR, fluid attenuated inversion recovery; MRI, magnetic resonance imaging.

Because prion disease is caused by an aberration of a normally expressed protein, there are many genetic aspects to the illness. In addition to playing a key role in genetic prion diseases, variations in *PRNP* sequences are also important for sporadic and acquired prion diseases. In this article, we will review how genetic polymorphisms affect all prion diseases. Additionally, we will review the genetic prion diseases and important information for clinicians. Finally, the burgeoning field of genetic approaches for experimental treatments of prion diseases will be discussed.

## Genetic associations in sporadic CJD

Sporadic CJD accounts for approximately 85% of all prion diseases. The exact cause of sCJD is unknown and there is no consistent evidence that suggests it is due to infectious transmission. sCJD incidence increases with age, hence sCJD could be due to the stochastic production and reduced clearance of PrP^D^. Alternatively, sCJD could be due to somatic mutations. In addition to age, *PRNP* polymorphisms have been associated with risk of developing sCJD, specifically those at codons 129 and 219.

### Codon 129

Polymorphic variation at codon 129 of *PRNP* affects risk of developing sCJD. Three polymorphic combinations can occur at codon 129: methionine-methionine (MM), methionine-valine (MV), and valine-valine (VV). In one analysis, the distribution of codon 129 polymorphisms in a healthy Caucasian population was 51% MV, 37% MM, and 12% VV (13). However, homozygosity is overrepresented in sCJD, especially methionine homozygosity (14). In contrast, methionine homozygosity at codon 129 is vastly overrepresented in normal Asian populations (>90%) and an initial Japanese study did not find an association between methionine homozygosity and the development of sCJD (15, 16). However, when the effects of the codon 219 polymorphism (see below) were accounted for in a multivariable logistic regression model, methionine homozygosity at codon 129 did increase the likelihood of developing sCJD in a Japanese population (17). A meta-analysis examining the association of codon 129 with sCJD also found that methionine homozygosity is a significant risk factor for the development of sCJD compared to heterozygosity (OR: 4.96, 95% CI: 3.48–7.08) (18). Homozygosity is likely a risk factor for sCJD due to sequence homogeneity in expressed prion proteins, thereby enabling a more efficient conversion of PrP^c^ to PrP^D^.

In addition to being a risk factor for sCJD, polymorphic variation at codon 129 also modifies the phenotypic expression of sCJD. Codon 129 heterozygotes have a longer mean survival time, followed by valine homozygotes and methionine homozygotes, respectively (19). Functional neurologic decline also follows this pattern with methionine homozygotes having the quickest functional decline and heterozygotes having the slowest (20). Because of its influence over disease duration, codon 129 polymorphism can be used in predictive models of survival in sCJD (21). When codon 129 polymorphism is combined with the prion protein type (e.g., type 1, type 2), as determined by western blot analyses, six molecular subtypes of sCJD can be defined (e.g., MM1, VV2, MV2). sCJD molecular subtypes differ in clinical presentation, age at onset, disease duration, diagnostic test results, and neuropathologic findings (6, 22–25) (Table 2). Unlike sCJD, most cases of variably protease sensitive prionopathy (VPSPr), another sporadic prion disease, are homozygous for valine at codon 129 (65%) (26). Patients affected by VPSPr, especially valine homozygotes, may also have a family history of other neurodegenerative conditions.

**Table 2:** Clinical characteristics of sporadic Creutzfeldt-Jakob disease (sCJD) molecular subtypes (6, 8, 23, 27).

**sCJD Subtype**	**Percentage of sCJD cases %**	**Mean Age at Onset (y)**	**Mean Duration (mo)**	**Clinical Presentation**	**PSWC on EEG (%)**	**CSF 14-3-3 positive (%)**	**RT-QuIC positive (%)**	**Brain MRI Positive (%)**
MM1	68	66	4	Classic CJD (cognitive impairment and ataxia), Heidenhain variant (visual presentation)	80	100	96	65–93
MM2^*^	2	64	16	Cognitive impairment and aphasia	0	75	78	70–95
MV1	4	62	5	Cognitive impairment and ataxia	71	89	81	80–93
MV2	9	59	17	Atypical dementia, ataxia, psychiatric symptoms	8	89	93	86–100
VV1	1	39	15	Cognitive impairment and aphasia	0	100	0	62–90
VV2	16	61	7	Oppenheimer-Brownell variant (ataxia)	7	100	96	53–84

M, methionine; V, valine; sCJD, sporadic Creutzfeldt-Jakob disease; PSWC, periodic sharp wave complexes; EEG, electroencephalogram; CSF, cerebrospinal fluid; MRI, magnetic resonance imaging.

* MM2 with neuronal loss/gliosis confined to the thalamus and/or olivary nuclei is referred to as sporadic fatal insomnia.

### Codon 219

In addition to codon 129, a polymorphism at codon 219 of *PRNP* is strongly protective for sCJD. This polymorphism only occurs in east and south Asian populations and accounts for approximately 14% and 8% of the general Japanese and Korean populations, respectively (16). Heterozygosity at codon 219 is rarely seen in sCJD (< 1% of cases) but is more common in acquired prion diseases (17).

### Other associations

Although most genetic risk for sCJD is associated with *PRNP*, genetic variation of other genes may also play a role. Genome wide association studies (GWAS) are often used to detect genetic factors that may affect diseases, and typically a large sample is required to achieve statistical significance. The rarity of human prion diseases poses a challenge for GWAS, and some studies have been limited by sample size (28–30). However, the largest GWAS performed to date included 5,208 cases of sCJD that were used to independently replicate findings. Three genes were associated with risk of sCJD; *PRNP, STX6*, and *GAL3ST1* (31). Genetic variation in *STX6* is also associated with progressive supranuclear palsy (PSP), a primary tauopathy. These findings suggest that changes to intracellular transport and sphingolipid metabolism may increase the risk of sCJD and other protein misfolding disorders. The age-associated risk of developing sCJD, possibility of non-*PRNP* gene effects on its development and/or clinical course, and dependence on a host-encoded protein for disease suggest that epigenetic factors may also play a role in disease pathogenesis (32). However, current research into epigenetic risk factors is preliminary and requires further study.

## Genetic associations in acquired prion diseases

Acquired prion disease occurs when exogenous prions are transmitted to an individual. Acquired prion diseases include kuru, iatrogenic CJD (iCJD), and variant (CJD). Prions can be transmitted through ingestion of prion contaminated food (i.e., kuru and vCJD) or transmitted through medical procedures such as reusing select cadaveric materials (i.e., pituitary gonadotropins, growth hormone, dura mater grafts, and corneal transplants) or contaminated instrumentation (i.e., EEG depth electrodes and neurosurgical instruments). Variant CJD is the only form of human prion disease that has demonstrated infectivity through blood products.

Like sCJD, codon 129 genotype affects susceptibility to acquired prion diseases as well as other characteristics of the illness. Homozygosity at codon 129 increases the risk of iCJD with homozygotes being over represented and the frequency of MM and VV codon 129 genotypes differing between countries (13, 33, 34). Codon 129 genotype also influences incubation periods. In general, homozygotes have a shorter incubation period compared to heterozygotes (34, 35). Methionine homozygosity was also a risk factor for acquiring kuru and was associated with shorter incubation periods (36). Interestingly, Kuru caused selection of the 129V allele, a more resistant genotype to kuru, when endocannabilism was active in Papua New Guinea (37). A kuru resistant polymorphism (G127V), only found in populations affected by kuru, has also been described in subjects who did not develop kuru, predominantly codon 129 methionine homozygotes (38, 39).

Much of what is known about iCJD and kuru was used to model public health estimates for vCJD. When vCJD was first identified and later found to be associated with eating beef contaminated with bovine spongiform encephalopathy (BSE), public health officials and government representatives looked to other acquired prion diseases to help predict health outcomes (40). Although there are important differences in vCJD, namely that it is a zoonotic illness caused by a non-human prion strain, there are many similarities to other acquired human prion diseases. All but one case of autopsy confirmed vCJD has been homozygous for methionine at codon 129 (41, 42). This finding suggests that methionine homozygotes are at greater risk of developing vCJD. Knowledge obtained from studying other acquired prion diseases demonstrate that codon 129 genotype also affects incubation period. Hence, one possibility is that we may see other cases of vCJD of different codon 129 genotypes (i.e., MV and VV) as the time from exposure lengthens. Preclinical studies that include a case of transfusion related vCJD and surveys of appendectomy specimens indicate that asymptomatic vCJD infectivity can occur in all codon 129 genotypes (43–45). One autopsy-confirmed symptomatic case of vCJD case was heterozygous at codon 129. Importantly, this heterozygous vCJD case did not meet the clinical criteria for vCJD and had a brain MRI that resembled sCJD. Knowledge from other prion diseases would suggest that codon 129 genotype could influence clinical phenotype and diagnostic test results. Thus, it is possible that without autopsy confirmation, vCJD cases that are not homozygous for methionine at codon 129 may be misdiagnosed as probable sCJD.

## Genetic prion diseases

Genetic prion disease causes 10–15% of all cases and is caused by pathogenic variations in *PRNP*. Three types of causal variants/mutations have been identified: missense mutations, insertions or deletions of an octapeptide region of the N-terminal domain, and non-sense mutations (stop codon). Over 60 different mutations have been described in the literature (46), the most common of which is E200K. Four mutations account for more than half of all genetic prion diseases (E200K, D178N, P102L, and A117V). Given the extensive number of *PRNP* mutations, a detailed discussion of each mutation is beyond the scope of this article. Rather, this article will focus on prevalent themes that are pertinent to clinical practice.

Genetic prion disease is typically broken into three broad clinicopathologic phenotypes: genetic CJD (gCJD), fatal familial insomnia (FFI), and Gerstmann-Straussler-Scheinker syndrome (GSS). These phenotypes differ in clinical phenomenology, diagnostic test results, and neuropathologic features. Although there are general associations between different mutations and these clinicopathologic phenotypes, phenotypic heterogeneity exists within mutations and in families that carry the same mutation. Given the historical classification of these diseases and the clinical approach of this review, the authors have chosen to present features of genetic prion disease by clinicopathologic phenotype.

### Genetic Creutzfeldt-Jakob disease (gCJD)

gCJD is the most common genetic prion disease and is mostly caused by missense mutations. E200K is the most common cause of gCJD worldwide. gCJD closely resembles the clinicopathologic features of sCJD. Clinical presentation is heterogenous, like sCJD, but typically includes cognitive impairment, ataxia, and myoclonus. Although variability exists between mutations and within mutations, disease onset typically occurs in midlife and illness duration is typically under 1 year. Diagnostic test results differ by mutation, but CSF RT-QuIC is frequently positive and brain MRIs are often suggestive of prion disease (6, 47). Periodic Sharp Wave Complexes (PSWCs) on electroencephalogram (EEG) and 14-3-3 proteins in CSF are sometimes observed in gCJD, but not as commonly as sCJD. Neuropathologic findings are similar to sCJD, though some mutation specific characteristics have been described (48).

### Fatal familial insomnia (FFI)

FFI is a clinicopathologic phenotype causes by the D178N mutation when codon 129M is in *cis* with the mutated allele. A gCJD phenotype is usually seen when D178N is in *cis* with 129V. FFI typically starts in midlife and disease duration is typically longer than 1 year (49). Clinical symptoms are characterized by early sleep disturbance, autonomic instability, and symptoms usually associated with CJD (i.e., cognitive impairment, ataxia, and myoclonus). Diagnosis can be difficult without a known family history given the low sensitivity of diagnostic tests typically used in prion diseases. PSWCs on EEG, CSF 14-3-3 proteins, and brain MRI findings are typically absent in FFI. Aside from genetic testing, which would be definitive, suggestive antemortem diagnostic tests include sleep architecture disruption on polysomnogram and thalamic hypometabolism on brain fluorodeoxyglucose positron emission tomography (FDG-PET) or single-photon emission computed tomography (SPECT). CSF RT-QuIC diagnostic sensitivity for FFI may differ depending on the population being tested as Caucasian cases are frequently negative and Asian cases are frequently positive (6, 49).

### Gerstmann-Straussler-Scheinker syndrome (GSS)

GSS is associated with a variety of missense and nonsense mutations (premature stop mutations) as well as some octapeptide repeat insertion mutations. Neuropathologic features of GSS include prion protein amyloidosis (e.g., amyloid plaques), neuronal loss, and glial proliferation in the cortex, deep gray nuclei, and cerebellum (50). Some forms of GSS have concomitant tau pathology. Western blot analyses demonstrate truncated protease resistant prion fragments, called internal fragments, that are often 7–8 kDa in size. Disease onset and duration is often mutation dependent but can sometimes occur as early as the 20 s and mean disease duration is ~5 years. Clinical symptoms can include early cerebellar dysfunction, parkinsonism, or frontotemporal dementia-like symptoms. PSWCs on EEG and 14-3-3 proteins in CSF are usually not detected in GSS. Positive CSF RT-QuIC and hyperintensity on brain MRIs are sometimes seen in some GSS mutations.

### Octapeptide repeat insertions and deletions

Insertions and deletions in the octapeptide region can also cause genetic prion disease with different clinicopathologic phenotypes. Octapeptide repeat insertion (OPRI) mutations can differ in the sequence and arrangement of repeats, but clinicopathologic phenotype is most influenced by the number of repeats (47). One octapeptide repeat insertion (OPRI) variants are considered benign and 2-OPRI may minimally increase risk of developing CJD (51). In general, penetrance increases with the number of repeats and clinicopathologic phenotypes exist on a spectrum where low repeat numbers (< 8) tend to demonstrate a CJD phenotype and higher repeat numbers (≥8) trend toward a GSS phenotype. Similarly, age at onset is inversely related to repeat number and illness duration correlates with the number of repeats. However, these are broad generalizations and there is considerable variability in these characteristics across repeat numbers. 1- and 2-octapeptide repeat deletions (OPRD) have also been described in the literature. 1-OPRD is considered a benign polymorphism, whereas 2-OPRD is considered a pathogenic variant. Because no reported cases of 2-OPRD had a family history of prion disease and only five cases have been detected through international prion disease surveillance centers, penetrance is likely low (47, 52).

### Stop codon mutations

Stop codon or nonsense *PRNP* mutations are typically associated with PrP amyloid angiopathy and demonstrate a wide array of clinical phenotypes. Many nonsense mutations cause non-specific progressive cognitive impairment or Alzheimer's-like dementia (Y145X, Y160X, Y226X). However, a unique phenotype has been described with Y163X, which causes systemic PrP amyloidosis resulting in diarrhea and autonomic neuropathy (53).

Whereas the majority of the clinicopathologic phenotype is influenced by the specific mutation, the codon 129 genotype is also important in genetic prion diseases. Perhaps the best example of this is the different phenotypes, gCJD and FFI, seen with the D178N mutation. The phenotype is determined by the codon 129 polymorphism located on the mutated allele. Hence, the D178N-129V haplotype is typically expressed as a gCJD phenotype whereas the D178N-129M haplotype usually results in a FFI phenotype. Although this is the most dramatic example, *PRNP* mutations are often described by haplotype because of frequent differences observed between codon 129 genotypes on the mutated allele. There is also evidence that the codon 129 genotype of the non-mutated allele may influence disease characteristics. For example, in FFI homozygotes have a shorter mean disease duration compared to heterozygotes (49).

Once thought to be fully penetrant, it is now recognized that genetic prion diseases have variable levels of penetrance. Extensive work conducted by Minikel and colleagues has provided the field with important information regarding different penetrance levels across *PRNP* mutations (46). Although there are mutations that are nearly completely penetrant (D178N, P102L), some mutations exhibit low penetrance such as V180I (1%) and V210I (10%). The most common genetic prion disease mutation, E200K, exhibits incomplete and age-associated penetrance with most mutation carriers developing illness with advancing age. Estimated penetrance of specific mutations is important information to convey to families affected by genetic prion disease and may influence an individual's choice to undergo asymptomatic genetic testing. Whenever possible, information regarding penetrance should be conveyed to families when a mutation is initially disclosed to the family. Most national prion disease surveillance centers can assist genetic counselors and clinicians with this information if needed.

Some genetic illnesses exhibit anticipation, when the age of disease onset commences at earlier ages with successive generations. Several studies reported on the possibility of anticipation in gCJD with the E200K mutation. However, an international study examining 217 individuals with the E200K mutation failed to detect anticipation across generations, attributing earlier reports to ascertainment bias (54). Because genetic prion diseases are not affected by factors associated with anticipation, such as germline expansion of unstable repeats and telomere shortening, there is no biological plausible explanation for such a phenomenon. Thus, anticipation is not likely to affect genetic prion diseases caused by other mutations either.

## Genetic testing

Genetic testing for *PRNP* variants can be done at autopsy or prior to the patient's death using blood, buccal swabs, or saliva. Although most cases of genetic prion diseases will have a family history of prion disease, this is not always the case. Exceptions can be due to low penetrance mutations, misdiagnoses of family members, death of mutation carriers prior to clinical onset of disease, misattributed parentage, or uninformed family history. Hence, genetic testing should always be considered to definitively rule out genetic forms of the disease, as well as to confirm diagnosis and type of prion disease (e.g., sporadic, genetic, variant, or novel prion disease). When conducting post-mortem genetic testing, the possibility of detecting a pathogenic genetic variant should always be considered and discussed with the family. At the time of autopsy consent, the legal next of kin should be asked whether they would like genetic information disclosed as part of the autopsy report. The family can defer receiving genetic information until a later date. The legal next of kin is encouraged to have discussions with all first-degree relatives who may be directly affected by genetic results. Some may wish to know this information, but some may not. Knowledge of others' wishes who will be directly impacted by the genetic results should be considered by the legal next of kin. Genetic counseling can be helpful to weigh the pros and cons of learning this information, how to approach the issue with other family members, and how to interpret results (55). Post-mortem examination results should be discussed with family members, in person whenever possible. If the clinician receiving the results has questions regarding them, they are encouraged to contact the testing facility for more information and guidance. If a pathogenic mutation is discovered, the clinician should have basic information regarding this mutation available for discussion. Such mutation-specific information includes typical clinical presentation, mean age of onset, duration, and most importantly, penetrance (if known) (56). Prion disease centers such as the National Prion Disease Pathology Surveillance Center (cjdsurveillance.com) and patient advocacy groups such as the CJD Foundation (cjdfoundation.org) can provide clinicians with up-to-date information and guide them through this process.

Predictive genetic testing is available to individuals with a family history of genetic prion disease, defined as a family member with a *PRNP* mutation or two or more family members diagnosed with prion disease. Patients may choose to undergo predictive genetic testing for a variety of reasons, including family planning, financial planning, willingness to participate in research, or for no specific reason aside from knowing their mutation status. Predictive genetic testing should be approached in a cautious and systematic manner given its implications in this uniformly fatal illness. Patients undergoing predictive genetic testing should receive genetic counseling prior to being tested. Genetic counseling should follow the Huntington disease protocol for predictive testing (HD protocol) (57). The HD protocol is a multi-step interdisciplinary process that ensures the patient has adequate information, support, and time to make the correct decision for themselves. The HD protocol involves genetic counseling and neurologic and psychiatric examinations prior to genetic testing. Following testing, much of the pre-testing information and assessments are repeated and results are disclosed in the presence of a support person. Information reviewed in this process includes knowledge regarding the mutation (clinical characteristics, mean age of onset, mean duration, penetrance), assisting the patient with examining why they desire this knowledge and what they plan on doing with it, and ensuring that they comprehend all implications of knowing their genetic status including concepts such as survivor's guilt in mutation-negative individuals. Pre-testing neurologic examinations are important to determine whether someone may be symptomatic since many patients have been found to develop symptoms shortly after receiving results (58). This suggest that prodromal symptoms may prompt some individuals to undergo genetic testing. Psychiatric screening is important to assess mental stability to receive results and risk of suicidality as there is a large amount of psychological burden in families affected by genetic prion disease (59). Individual preferences and needs vary, and a modified version of the HD protocol may be considered for some individuals (60). Modifications may include having different clinicians perform some of the assessments if access to some specialties are limited or improving access to knowledgeable counselors using telemedicine. Genetic testing is not conducted on minors unless there is valid concern that the individual may be symptomatic, which would rarely occur in genetic prion disease.

One reason for undergoing predictive genetic testing is family planning. There are several options available for mutation carriers who would like to have children that are not at risk of carrying the mutation. Adoption is one option but removes the possibility of biological children. There are two primary options for having biological children without the mutation. Genetic testing can be performed *in utero* and may change pregnancy management should the fetus carry the mutation. However, this approach can raise moral, ethical, and legal issues, and may not be suitable for all patients. An alternative approach is preimplantation genetic diagnosis (PGD) with *in vitro* fertilization which selectively implants embryos that do not carry a mutation (61). PGD can even be performed if the parent does not wish to know their own mutation status.

## Genetic approaches to treatment

There are several possible treatment targets in prion disease and genetic modifying approaches are potentially promising (62). Prior clinical trials have mainly focused on reducing conversion of PrP^c^ to PrP^D^. Experimental therapies using this approach have largely failed in human clinical trials (e.g., quinacrine, doxycycline, pentosan polysulfate). Other possible avenues for treatment include reducing toxicity of PrP^D^, which is complicated by the fact that this mechanism is poorly understood, or increasing clearance of PrP^D^. Because the expression of normal host PrP^c^ is required for prion disease, targeting the substrate that underlies prion disease is an alternative approach that holds promise for all forms of prion disease.

Reducing the amount of PrP^c^ removes a necessary requirement for prion disease and is potentially achievable with current genetic modifying techniques. The complete function of PrP^c^ is poorly understood, but it is highly conserved across species. *PRNP* knock out mice are phenotypically normal with some slight electrophysiologic and circadian rhythm changes (63, 64). Complete ablation of the prion protein caused chronic demyelinating polyneuropathy in mice, suggesting that it has some role in myelin maintenance (65). Importantly, older individuals with heterozygous loss of *PRNP* function have been identified and were devoid of overt neurologic illness (46). These data suggest that affecting normal production of PrP^c^ would not likely be catastrophic, but less severe effects are unknown.

Genetic approaches to treatment would primarily be designed to reduce the production of PrP^c^, not eliminate it. Theoretically, any genetic modification that results in reduced PrP^c^ would be expected to demonstrate a possible treatment effect. A variety of genetic approaches to treatment could achieve this goal such as gene editing (CRISPR/Cas9) and a variety of genetic silencing techniques. Although CRISPR/Cas9 has been examined in some prion disease models, it has not been extensively studied in prion disease treatment models and has some limitations in treating post-natal genetic neurologic diseases (66–68). Silencing techniques, including antisense oligonucleotides (ASOs) and RNA interference (RNAi), have been most the most studied genetic approaches in preclinical models of prion disease. Engineered ASOs bind to complementary RNA of the prion protein, leading to its degradation and overall decrease in PrP^c^ production (69). ASOs do not cross the blood-brain barrier and need to be administered *via* intrathecal infusion. Mice that were intracerebrally infected with prions were administered ASOs every 2–3 months and demonstrated prolonged illness durations by 61–98% compared to untreated mice (70). Survival was prolonged by 55% in mice that were given one injection late in the incubation period. ASOs also prolonged survival when administered to mice who were already symptomatic (71). RNAi reduces target protein by facilitating degradation of mRNA or through silencing gene expression by interfering with protein translation. RNAi has successfully reduced PrP^c^ in a variety of species (72). RNAi has also been shown to prolong survival in some prion inoculated mice. Total prion protein can be reliably measured in human CSF to assess target engagement and measure its reduction with treatment (27). As baseline CSF prion protein levels are lower in mutation carriers and differ between mutations, measurement of the relative reduction of total prion protein levels would be required to accurately assess target engagement (73).

Although genetic approaches to treatment seems promising in prion disease, there remain several challenges. Because of the variety of genetic prion disease mutations and the heterogenous age at clinical onset of these mutations, evaluating a treatment's effect on asymptomatic pathogenic variant carriers by measuring time until illness onset is impractical (74). Surrogate markers will be required to study these treatments more efficiently in genetic mutation carriers. Total prion protein CSF levels could serve as a measure of target engagement and as a disease-specific biomarker. Other markers of neurodegeneration, such as neurofilament light chain (NfL) may also hold promise in measuring treatment effects prior to overt phenoconversion (75). Animal models suggest that treatments could be beneficial to symptomatic patients as well. However, successfully treating symptomatic prion disease patients will require early diagnosis to have a chance of producing a clinically meaningful effect.

## Summary

Prion diseases are caused by a disease-associated form of the prion protein that causes a rapid and fatal neurodegenerative illness. Although there are three types of prion diseases (sporadic, genetic, and acquired), all forms are affected by *PRNP* as the production of PrP^c^ is necessary for disease. *PRNP* can contain pathogenic variants/mutations that result in various forms of genetic prion disease that differ in penetrance, clinical phenomenology, age at onset, disease duration, and diagnostic test results. However, all prion diseases are also affected by *PRNP* polymorphisms, with the polymorphic codon 129 having the strongest influence across all prion diseases. Genetic testing should always be discussed with families affected by prion disease, regardless of the presence of a family history. Predictive genetic testing is available for at-risk individuals and should be performed using the HD protocol. There are several options available to at-risk individuals who desire to have biological children free from *PRNP* mutations. Predictive genetic testing may increase as individuals may want to participate in upcoming clinical trials. Genetic approaches to treatment may be more applicable to prion diseases than other neurodegenerative conditions and could potentially be used to prevent or delay clinical onset in mutation carriers and treat patients with symptomatic disease. Genetic therapies aim to reduce PrP^c^, which removes a necessary substrate for prion disease pathogenesis. More work is needed to evaluate how genetic therapies may impact human prion disease.

## Author contributions

BA performed a review of the literature and wrote/edited the manuscript. SS and ME provided expertise and edited the manuscript. All authors contributed to the article and approved the submitted version.

## Funding

This work was partially funded by CDC grant (NU38CK000480).

## Conflict of interest

Author BA has received research funding from CDC, NIH, CJD Foundation, Ionis, and Alector. He has provided consultation for Ionis, Acadia and Sangamo, as well as being the Medical Director for the CJD Foundation. Author SS has received research funding from the CJD Foundation. The remaining author declares that the research was conducted in the absence of any commercial or financial relationships that could be construed as a potential conflict of interest.

## Publisher's note

All claims expressed in this article are solely those of the authors and do not necessarily represent those of their affiliated organizations, or those of the publisher, the editors and the reviewers. Any product that may be evaluated in this article, or claim that may be made by its manufacturer, is not guaranteed or endorsed by the publisher.

## References

[1] Prusiner SB. Novel proteinaceous infectious particles cause scrapie. Science. (1982) 216:136–44. 10.1126/science.6801762 6801762

[2] Bueler H Aguzzi A Sailer A Greiner RA Autenried P Aguet M. Mice devoid of PrP are resistant to scrapie. Cell. (1993) 73:1339–47. 10.1016/0092-8674(93)90360-3 8100741

[3] Sailer A Büeler H Fischer M Aguzzi A Weissmann C. No propagation of prions in mice devoid of PrP. Cell. (1994) 77:967–8. 10.1016/0092-8674(94)90436-7 7912659

[4] Klug GMJA Wand H Simpson M Boyd A Law M Masters CL. Intensity of human prion disease surveillance predicts observed disease incidence. J Neurol Neurosurg Psychiatry. (2013) 84:1372–7. 10.1136/jnnp-2012-304820 23965290

[5] Maddox RA Person MK Blevins JE Abrams JY Appleby BS Schonberger LB. Prion disease incidence in the United States, 2003–2015. Neurology. (2020) 94:e153–7. 10.1212/WNL.0000000000008680 31757870

[6] Rhoads DD Wrona A Foutz A Blevins JE Glisic K Person MK. Diagnosis of Prion diseases by RT-QuIC results in improved surveillance. Neurology. (2020) 95:e1017–26. 10.1212/WNL.0000000000010086 32571851

[7] Figgie MP Appleby BS. Clinical use of improved diagnostic testing for detection of prion disease. Viruses. (2021) 13:789. 10.3390/v13050789 33925126 PMC8146465

[8] Bizzi A Pascuzzo R Blevins JE Grisoli M Lodi R Moscatelli M. New criterion for detecting prion disease with diffusion magnetic resonance imaging: diagnostic performance and comparison with cerebrospinal fluid tests in a large autopsy cohort. JAMA Neurol. (2020) 132:2659–68. 10.1001/jamaneurol.2020.1319 32478816 PMC7265127

[9] Brown P Brandel JP Preese M Sato T. Iatrogenic Creutzfeldt-Jakob disease: the waning of an era. Neurology. (2006) 67:389–93. 10.1212/01.wnl.0000231528.65069.3f 16855204

[10] Zeidler M Sellar RJ Collie DA Knight R Stewart G Macleod MA. The pulvinar sign on magnetic resonance imaging in variant Creutzfeldt-Jakob disease. Lancet. (2000) 355:1412–8. 10.1016/S0140-6736(00)02140-1 10791525

[11] Zerr I Kallenberg K Summers DM Romero C Taratuto A Heinemann U. Updated clinical diagnostic criteria for sporadic Creutzfeldt Jakob disease. Brain. (2009) 132:2659–68. 10.1093/brain/awp191 19773352 PMC2759336

[12] Centers for Disease Control and Prevention. Diagnostic Criteria: CDC's Diagnostic Criteria for Creutzfeldt-Jakob Disease (CJD), 2018. Diagnostic Criteria: CDC's Diagnostic Criteria for Creutzfeldt-Jakob Disease (CJD), 2018. (2019). Available online at: https://www.cdc.gov/prions/cjd/diagnostic-criteria.html (accessed August 29, 2019).

[13] Collinge J Palmer MS Dryden AJ. Genetic predisposition to iatrogenic Creutzfeldt-Jakob disease. Lancet. (1991) 337:1441–2. 10.1016/0140-6736(91)93128-V 1675319

[14] Palmer MS Dryden AJ Hughes JT Collinge J. Homozygous prion protein genotype predisposes to sporadic Creutzfeldt–Jakob disease. Nature. (1991) 352:340–2. 10.1038/352340a0 1677164

[15] Doh-ura K Kitamoto T Sakaki Y Tateishi J. CJD discrepancy. Nature. (1991) 353:801–2. 10.1038/353801b0 1682813

[16] Jeong B-H Nam J-H Lee Y-J Lee K-H Jang M-K Carp RI. Polymorphisms of the prion protein gene (PRNP) in a Korean population. J Hum Genet. (2004) 49:319–24. 10.1007/s10038-004-0150-7 15148589

[17] Kosami K Ae R Hamaguchi T Sanjo N Tsukamoto T Kitamoto T. Methionine homozygosity for PRNP polymorphism and susceptibility to human prion diseases. J Neurol Neurosurg Psychiatry. (2022) 93:779–84. 10.1136/jnnp-2021-328720 35387866

[18] Kim Y-C Jeong B-H. The first meta-analysis of the M129V Single-Nucleotide Polymorphism (SNP) of the Prion Protein Gene (PRNP) with Sporadic Creutzfeldt–Jakob Disease. Cells. (2021) 10:3132. 10.3390/cells10113132 34831353 PMC8618741

[19] Pocchiari M Puopolo M Croes EA Budka H Gelpi E Collins S. Predictors of survival in sporadic Creutzfeldt-Jakob disease and other human transmissible spongiform encephalopathies. Brain. (2004) 127:2348–59. 10.1093/brain/awh249 15361416

[20] Mead S Burnell M Lowe J Thompson A Lukic A Porter M-C. Clinical trial simulations based on genetic stratification and the natural history of a functional outcome measure in Creutzfeldt-Jakob Disease. JAMA Neurol. (2016) 73:447–55. 10.1001/jamaneurol.2015.4885 26902324 PMC5701731

[21] Llorens F Rübsamen N Hermann P Schmitz M Villar-Piqué A Goebel S. A prognostic model for overall survival in sporadic Creutzfeldt-Jakob disease. Alzheimers Dement. (2020) 16:1438–47. 10.1002/alz.12133 32614136

[22] Parchi P Castellani R Capellari S Ghetti B Young K Chen SG. Molecular basis of phenotypic variability in sporadic Creutzfeldt-Jakob disease. Ann Neurol. (1996) 39:767–78. 10.1002/ana.410390613 8651649

[23] Parchi P Giese A Capellari S Brown P Schulz-Schaeffer W Windl O. Classification of sporadic Creutzfeldt-Jakob disease based on molecular and phenotypic analysis of 300 subjects. Ann Neurol. (1999) 46:224–33. 10443888

[24] Appleby BS Rhoads DD Mente K Cohen ML. A practical primer on Prion pathology. J Neuropathol Exp Neurol. (2018) 77:346–52. 10.1093/jnen/nly019 29608707

[25] Bizzi A Pascuzzo R Blevins J Moscatelli MEM Grisoli M Lodi R. Subtype diagnosis of sporadic Creutzfeldt–Jakob disease with diffusion magnetic resonance imaging. Ann Neurol. (2021) 89:560–72. 10.1002/ana.25983 33274461 PMC7986086

[26] Notari S Appleby BS Gambetti P. “Variably protease-sensitive prionopathy,” *Handbook of Clinical Neurology*. Amsterdam: Elsevier (2018). p. 175–190. 10.1016/B978-0-444-63945-5.00010-6 29887135

[27] Vallabh SM Nobuhara CK Llorens F Zerr I Parchi P Capellari S. Prion protein quantification in human cerebrospinal fluid as a tool for prion disease drug development. Proc Natl Acad Sci U S A. (2019) 116:7793–8. 10.1073/pnas.1901947116 30936307 PMC6475435

[28] Sanchez-Juan P Bishop MT Croes EA Knight RS Will RG van Duijn CM. A polymorphism in the regulatory region of PRNP is associated with increased risk of sporadic Creutzfeldt-Jakob disease. BMC Med Genet. (2011) 12:73. 10.1186/1471-2350-12-73 21600043 PMC3114709

[29] Mead S Poulter M Uphill J Beck J Whitfield J Webb TE. Genetic risk factors for variant Creutzfeldt–Jakob disease: a genome-wide association study. Lancet Neurol. (2009) 8:57–66. 10.1016/S1474-4422(08)70265-5 19081515 PMC2643048

[30] Poleggi A van der Lee S Capellari S Puopolo M Ladogana A Pascali ED. Age at onset of genetic (E200K) and sporadic Creutzfeldt-Jakob diseases is modulated by the *CYP4X1* gene. J Neurol Neurosurg Psychiatry. (2018) 89:1243–9. 10.1136/jnnp-2018-318756 30032116

[31] Jones E Hummerich H Vire E Uphill J Dimitriadis A Speedy H. Identification of novel risk loci and causal insights for sporadic Creutzfeldt-Jakob disease: a genome-wide association study. Lancet Neurol. (2020) 19:840–8. 10.1016/S1474-4422(20)30273-8 32949544 PMC8220892

[32] Viré EA Mead S. Gene expression and epigenetic markers of prion diseases. Cell Tissue Res. (2022) 10.1007/s00441-022-03603-2 35307791 PMC10113299

[33] Deslys JP Marce D Dormont D. Similar genetic susceptibility in iatrogenic and sporadic Creutzfeldt-Jakob disease. J Gen Virol. (1994) 75:23–7. 10.1099/0022-1317-75-1-23 8113733

[34] Brown P Brandel J-P Sato T Nakamura Y MacKenzie J Will RG. Iatrogenic Creutzfeldt-Jakob disease, final assessment. Emerg Infect Dis. (2012) 18:901–7. 10.3201/eid1806.120116 22607808 PMC3358170

[35] d'Aignaux JH Costagliola D Maccario J de Villemeur TB Brandel JP Deslys JP. Incubation period of Creutzfeldt-Jakob disease in human growth hormone recipients in France. Neurology. (1999) 53:1197–201. 10.1212/WNL.53.6.1197 10522872

[36] Lee HS Brown P Cervenáková L Garruto RM Alpers MP Gajdusek DC. Increased susceptibility to Kuru of carriers of the PRNP 129 methionine/methionine genotype. J Infect Dis. (2001) 183:192–6. 10.1086/317935 11120925

[37] Mead S Whitfield J Poulter M Shah P Uphill J Beck J. Genetic susceptibility, evolution and the kuru epidemic. Philos Trans R Soc B Biol Sci. (2008) 363:3741–6. 10.1098/rstb.2008.0087 18849290 PMC2576515

[38] Mead S Whitfield J Poulter M Shah P Uphill J Campbell T. A novel protective Prion protein variant that colocalizes with Kuru exposure. N Engl J Med. (2009) 361:2056–65. 10.1056/NEJMoa0809716 19923577

[39] Asante EA Smidak M Grimshaw A Houghton R Tomlinson A Jeelani A. A naturally occurring variant of the human prion protein completely prevents prion disease. Nature. (2015) 522:478–81. 10.1038/nature14510 26061765 PMC4486072

[40] Will R Ironside J. A new variant of Creutzfeldt-Jakob disease in the UK. Lancet. (1996) 347:921–5. 10.1016/S0140-6736(96)91412-9 8598754

[41] Bishop MT Pennington C Heath CA Will RG Knight RSG. PRNP variation in UK sporadic and variant Creutzfeldt Jakob disease highlights genetic risk factors and a novel non-synonymous polymorphism. BMC Med Genet. (2009) 10:146. 10.1186/1471-2350-10-146 20035629 PMC2806268

[42] Mok T Jaunmuktane Z Joiner S Campbell T Morgan C Wakerley B. Variant Creutzfeldt–Jakob disease in a patient with Heterozygosity at PRNP Codon 129. N Engl J Med. (2017) 376:292–4. 10.1056/NEJMc1610003 28099827

[43] Peden AH Head MW Diane LR Jeanne EB James WI. Preclinical vCJD after blood transfusion in a PRNP codon 129 heterozygous patient. Lancet. (2004) 364:527–9. 10.1016/S0140-6736(04)16811-6 15302196

[44] Gill ON Spencer Y Richard-Loendt A Kelly C Dabaghian R Boyes L. Prevalent abnormal prion protein in human appendixes after bovine spongiform encephalopathy epizootic: large scale survey. BMJ. (2013) 347:f5675. 10.1136/bmj.f5675 24129059 PMC3805509

[45] Gill ON Spencer Y Richard-Loendt A Kelly C Brown D Sinka K. Prevalence in Britain of abnormal prion protein in human appendices before and after exposure to the cattle BSE epizootic. Acta Neuropathol. (2020) 139:9650976. 10.1007/s00401-020-02153-7 32232565 PMC7244468

[46] Minikel EV Vallabh SM Lek M Estrada K Samocha KE Sathirapongsasuti JF. Quantifying prion disease penetrance using large population control cohorts. Sci Transl Med. (2016) 8:322ra9. 10.1126/scitranslmed.aad5169 26791950 PMC4774245

[47] Ladogana A Kovacs GG. Genetic Creutzfeldt-Jakob disease. Handb Clin Neurol. (2018) 153:219–42. 10.1016/B978-0-444-63945-5.00013-1 29887139

[48] Baiardi S Rossi M Mammana A Appleby BS Barria MA Calì I. Phenotypic diversity of genetic Creutzfeldt–Jakob disease: a histo-molecular-based classification. Acta Neuropathol. (2021) 142:707–8. 10.1007/s00401-021-02350-y 34324063 PMC8423680

[49] Cracco L Appleby BS Gambetti P. Fatal familial insomnia and sporadic fatal insomnia. Handb Clin Neurol. (2018) 153:271–99. 10.1016/B978-0-444-63945-5.00015-5 29887141

[50] Ghetti B Piccardo P Zanusso G. Dominantly inherited prion protein cerebral amyloidoses - a modern view of Gerstmann-Sträussler-Scheinker. Handb Clin Neurol. (2018) 153:243–69. 10.1016/B978-0-444-63945-5.00014-3 29887140

[51] Brennecke N Cali I Mok T Speedy H Genomics England Research Consortium Hosszu L. Characterization of Prion Disease Associated with a Two-Octapeptide Repeat Insertion. Viruses. (2021) 13:1794. 10.3390/v13091794 34578375 PMC8473248

[52] Kim M-O Takada LT Wong K Forner SA Geschwind MD. Genetic PrP Prion Diseases. Cold Spring Harb Perspect Biol. (2018) 10:a033134. 10.1101/cshperspect.a033134 28778873 PMC5932589

[53] Mead S Gandhi S Beck J Caine D Gallujipali D Carswell C. A novel Prion disease associated with diarrhea and autonomic neuropathy. N Engl J Med. (2013) 369:1904–14. 10.1056/NEJMoa1214747 24224623 PMC3863770

[54] Minikel EV Zerr I Collins SJ Ponto C Boyd A Klug G. Ascertainment bias causes false signal of anticipation in genetic prion disease. Am J Hum Genet. (2014) 95:371–82. 10.1016/j.ajhg.2014.09.003 25279981 PMC4185115

[55] Clift K Guthrie K Klee EW Boczek N Cousin M Blackburn P. Familial Creutzfeldt-Jakob disease: case report and role of genetic counseling in post mortem testing. Prion. (2016) 10:502–6. 10.1080/19336896.2016.1254858 27929804 PMC5161295

[56] Stephen CD Schaefer PW Appleby BS Oakley DH. Case 5-2022: a 65-year-old woman with rapidly progressive weakness in the right arm and recurrent falls. N Engl J Med. (2022) 386:674–87. 10.1056/NEJMcpc2115852 35172059

[57] Goldman JS. Genetic testing and counseling in the diagnosis and management of young-onset dementias. Psychiatr Clin North Am. (2015) 38:295–308. 10.1016/j.psc.2015.01.008 25998117

[58] Owen J Beck J Campbell T Adamson G Gorham M Thompson A. Predictive testing for inherited prion disease: report of 22 years experience. Eur J Hum Genet. (2014) 22:1351–6. 10.1038/ejhg.2014.42 24713662 PMC4091984

[59] Schwartz M Brandel J-P Babonneau ML Boucher C Schaerer E Haik S. Genetic testing in Prion disease: psychological consequences of the decisions to know or not to know. Front Genet. (2019) 10:895. 10.3389/fgene.2019.00895 31616476 PMC6764331

[60] Goldman JS Vallabh SM. Genetic counseling for prion disease: updates and best practices. Genetics in Medicine. (2022) [In Press]: 10.1016/j.gim.2022.06.003 35819418

[61] Meiner V Weinberg N Safran A Israela L Sagi M Rosenmann H. Preimplantation exclusion of embryos at risk for prion diseases. Neurology. (2006) 66:607–8. 10.1212/01.WNL.0000197984.03391.9B 16505327

[62] Appleby BS Connor A Wang H. Therapeutic strategies for prion disease: a practical perspective. Curr Opin Pharmacol. (2019) 44:15–9. 10.1016/j.coph.2018.11.006 30508662

[63] Colling SB Collinge J Jefferys JGR. Hippocampal slices from prion protein null mice: disrupted Ca2+-activated K+ currents. Neurosci Lett. (1996) 209:49–52. 10.1016/0304-3940(96)12596-9 8734907

[64] Tobler I Gaus SE Deboer T Achermann P Fischer M Rülicke T. Altered circadian activity rhythms and sleep in mice devoid of prion protein. Nature. (1996) 380:639–42. 10.1038/380639a0 8602267

[65] Bremer J Baumann F Tiberi C Wessig C Fischer H Schwarz P. Axonal prion protein is required for peripheral myelin maintenance. Nat Neurosci. (2010) 13:310–8. 10.1038/nn.2483 20098419

[66] Kaczmarczyk L Mende Y Zevnik B Jackson WS. Manipulating the prion protein gene sequence and expression levels with CRISPR/Cas9. PLoS ONE. (2016) 11:e0154604. 10.1371/journal.pone.0154604 27128441 PMC4851410

[67] Castle AR Wohlgemuth S Arce L Westaway D. Investigating CRISPR/Cas9 gene drive for production of disease-preventing prion gene alleles. PLoS ONE. (2022) 17:e0269342. 10.1371/journal.pone.0269342 35671288 PMC9173614

[68] Wong PK Cheah FC Syafruddin SE Mohtar MA Azmi N Ng PY Chua EW. CRISPR gene-editing models geared toward therapy for hereditary and developmental neurological disorders. Front Pediatr. (2021) 9:592571. 10.3389/fped.2021.592571 33791256 PMC8006930

[69] Vallabh SM Minikel EV Schreiber SL Lander ES. Towards a treatment for genetic prion disease: trials and biomarkers. Lancet Neurol. (2020) 19:361–8. 10.1016/S1474-4422(19)30403-X 32199098

[70] Raymond GJ Zhao HT Race B Raymond LD Williams K Swayze EE. Antisense oligonucleotides extend survival of prion-infected mice. JCI Insight. (2019) 5:e131175. 10.1172/jci.insight.131175 31361599 PMC6777807

[71] Minikel EV Zhao HT Le J O'Moore J Pitstick R Graffam S. Prion protein lowering is a disease-modifying therapy across prion disease stages, strains and endpoints. Nucleic Acids Res. (2020) 48:10615–31. 10.1093/nar/gkaa616 32776089 PMC7641729

[72] White MD Mallucci GR. Therapy for prion diseases. Prion. (2009) 3:121–8. 10.4161/pri.3.3.9289 19597349 PMC2802775

[73] Mortberg MA Zhao HT Reidenbach AG Gentile JE Kuhn E O'Moore J. PrP concentration in the central nervous system: regional variability, genotypic effects, and pharmacodynamic impact. JCI Insight. (2022) 7:e156532. 10.1172/jci.insight.156532 35133987 PMC8986079

[74] Minikel EV Vallabh SM Orseth MC Brandel J-P Ha S Capellari S. Age at onset in genetic prion disease and the design of preventive clinical trials. Neurology. (2019) 93:e125–34. 10.1212/WNL.0000000000007745 31171647 PMC6656649

[75] Thompson AGB Anastasiadis P Druyeh R Whitworth I Nayak A Nihat A. Evaluation of plasma tau and neurofilament light chain biomarkers in a 12-year clinical cohort of human prion diseases. Mol Psychiatry. (2021) 26:5955–66. 10.1038/s41380-021-01045-w 33674752 PMC8758487

